# Editorial Comment: Efficacy and Safety of Complete Intraureteral Stent Placement versus Conventional Stent Placement in Relieving Ureteral Stent Related Symptoms: A Randomized, Prospective, Single Blind, Multicenter Clinical Trial

**DOI:** 10.1590/S1677-5538.IBJU.2020.02.03

**Published:** 2020-01-10

**Authors:** Alexandre Danilovic

**Affiliations:** 1 Hospital das Clínicas Faculdade de Medicina USP São PauloSP Brasil Serviço de Urologia, Hospital das Clínicas da Faculdade de Medicina da USP - HCFMUSP, São Paulo, SP, Brasil

Yoshida T ^1,2^, Inoue T ^3,2^, Taguchi M ^1,2^, Matsuzaki T ^3,2^, Murota T ^3,2^, Kinoshita H ^2^, Matsuda T ^2^

^1^ Department of Urology and Andrology, Kori Hospital, Kansai Medical University , Osaka , Japan;^2^ Department of Urology and Andrology, Kansai Medical University , Osaka , Japan;^3^ Department of Urology and Andrology, General Medical Center, Kansai Medical University , Osaka , Japan

J Urol. 2019 Jul;202(1):164-170

**DOI:** 10.1097/JU.0000000000000196 | **ACCESS:** 10.1097/JU.0000000000000196

## COMMENT

Ureteral stent related symptoms are among the most troublesome problems for the attending urologist. Stent design, positioning, material and size of the stent have been extensively studied (1).

This is a high quality study coming from Japan to investigate a novel concept in ureteral stent positioning to relieve stent related symptoms. The authors randomized patients who required ureteral stent after ureteroscopy into complete intraureteral or conventional stent placement group. Symptoms scores and total amount of analgesics administered were significantly lower in the intraureteral group. There was no difference in the complication rate between groups.

This innovative and ready to use stent placement technique should be tested with different populations.with a larger long-term URI trend in view of the popularity of the technique. There is a need for specific cancer survival studies among the different techniques.
